# Fear of COVID-19 and Perceived COVID-19 Infectability Supplement Theory of Planned Behavior to Explain Iranians’ Intention to Get COVID-19 Vaccinated

**DOI:** 10.3390/vaccines9070684

**Published:** 2021-06-22

**Authors:** Rafat Yahaghi, Safie Ahmadizade, Razie Fotuhi, Elham Taherkhani, Mehdi Ranjbaran, Zeinab Buchali, Robabe Jafari, Narges Zamani, Azam Shahbazkhania, Hengame Simiari, Jalal Rahmani, Nahid Yazdi, Hashem Alijani, Leila Poorzolfaghar, Fatemeh Rajabi, Chung-Ying Lin, Anders Broström, Mark D. Griffiths, Amir H. Pakpour

**Affiliations:** 1 Social Determinants of Health Research Center, Research Institute for Prevention of Non-Communicable Diseases, Qazvin University of Medical Sciences, Qazvin 3419759811, Iran; yahaghirafat@yahoo.com (R.Y.); ahmadizadehn@yahoo.com (S.A.); m.r.a.mafi@gmail.com (R.F.); Etaherkhani.mid1@gmail.com (E.T.); mehdiranjbaran90@yahoo.com (M.R.); bochalizeinab@gmail.com (Z.B.); ro.midewife@yahoo.com (R.J.); Zamani.ee@gmail.com (N.Z.); Aazam.shahbazkhani@gmali.com (A.S.); Hengame.Simiari@gmail.com (H.S.); Jrahmani49@gmail.com (J.R.); n.yazdi@qums.ac.ir (N.Y.); hs.alijani@gmail.com (H.A.); Poorzolfaghar6565@gmail.com (L.P.); Fatemeh_rajabi85@yahoo.com (F.R.); 2 Institute of Allied Health Sciences, College of Medicine, National Cheng Kung University, Tainan 70101, Taiwan; 3 Department of Occupational Therapy, College of Medicine, National Cheng Kung University, Tainan 70101, Taiwan; 4 Department of Public Health, College of Medicine, National Cheng Kung University Hospital, National Cheng Kung University, Tainan 70101, Taiwan; 5 Department of Nursing, School of Health and Welfare, Jönköping University, SE-55111 Jönköping, Sweden; anders.brostrom@ju.se; 6 Department of Clinical Neurophysiology, Linköping University Hospital, SE-58183 Linköping, Sweden; 7 International Gaming Research Unit, Psychology Department, Nottingham Trent University, Nottingham NG1 4FQ, UK; mark.griffiths@ntu.ac.uk

**Keywords:** COVID-19, fear, Iran, perceived infectability, theory of planned behavior, vaccine

## Abstract

One of the most efficient methods to control the high infection rate of the coronavirus disease 2019 (COVID-19) is to have a high coverage of COVID-19 vaccination worldwide. Therefore, it is important to understand individuals’ intention to get COVID-19 vaccinated. The present study applied the Theory of Planned Behavior (TPB) to explain the intention to get COVID-19 vaccinated among a representative sample in Qazvin, Iran. The TPB uses psychological constructs of attitude, subjective norm, and perceived behavioral control to explain an individual’s intention to perform a behavior. Fear and perceived infectability were additionally incorporated into the TPB to explain the intention to get COVID-19 vaccinated. Utilizing multistage stratified cluster sampling, 10,843 participants (4092 males; 37.7%) with a mean age of 35.54 years (SD = 12.00) completed a survey. The survey assessed TPB constructs (including attitude, subjective norm, perceived behavioral control, and intention related to COVID-19 vaccination) together with fear of COVID-19 and perceived COVID-19 infectability. Structural equation modeling (SEM) was performed to examine whether fear of COVID-19, perceived infectability, and the TPB constructs explained individuals’ intention to get COVID-19 vaccinated. The SEM demonstrated satisfactory fit (comparative fit index = 0.970; Tucker-Lewis index = 0.962; root mean square error of approximation = 0.040; standardized root mean square residual = 0.050). Moreover, perceived behavioral control, subjective norm, attitude, and perceived COVID-19 infectability significantly explained individuals’ intention to get COVID-19 vaccinated. Perceived COVID-19 infectability and TPB constructs were all significant mediators in the relationship between fear of COVID-19 and intention to get COVID-19 vaccinated. Incorporating fear of COVID-19 and perceived COVID-19 infectability effectively into the TPB explained Iranians’ intention to get COVID-19 vaccinated. Therefore, Iranians who have a strong belief in Muslim religion may improve their intention to get COVID-19 vaccinated via these constructs.

## 1. Introduction 

Due to the high infection rate and serious health consequences of infection [1,2,3,4], the coronavirus disease 2019 (COVID-19) was announced as a pandemic by the World Health Organization (WHO) on 11 March 2020 [5]. Apart from psychological and physical health, almost all aspects of human life have been affected, including (but not limited) to financial activities, social interaction, education, and occupation [6]. In order to overcome the difficulties caused by the COVID-19 pandemic on human lives (over 172 million confirmed cases and over 3.7 million deaths worldwide [https://covid19.who.int/, accessed on 7 June 2021]; nearly three million confirmed cases and over 80,000 deaths in Iran as of 7 June 2021 [https://covid19.who.int/region/emro/country/ir, accessed on 7 June 2021]). COVID-19 vaccination has been rapidly developed with more than 60 candidates under clinical examination [7]. Moreover, over three million vaccine doses had been administered in Iran as of 23 May 2021 [https://covid19.who.int/region/emro/country/ir, accessed on 7 June 2021].

Although the progress of COVID-19 vaccination appears promising (e.g., measles vaccine programs launched by Gavi have reduced measles-caused deaths with a reduction of more than a 95% [8]), issues concerning vaccination uptake have long existed because some individuals distrust vaccination, especially those who believe in some advocacy from online sources and skeptical communities [8,9]. Indeed, alleged consequences from the administration of some vaccines such as autism development have been claimed, even though there is no clinical evidence to support such a notion [10]. This distrust may therefore also be applied to COVID-19 vaccination and may subsequently hamper the speed of COVID-19 vaccination uptake worldwide. Vaccine hesitancy has been listed as one of the top ten global health threats, particularly as a consequence of vaccine misinformation in social media. In addition, individuals may not want to have to endure short-term adverse reactions, such as having a high fever and/or sore arms caused by the vaccines [10]. As a result, it is important for healthcare providers and policymakers to understand key factors explaining an individual’s intention to get COVID-19 vaccinated. More specifically, widespread COVID-19 vaccination alongside herd immunity may reduce the infection rate and mortality of COVID-19.

Using a theory can efficiently and rapidly identify key psychological factors that explain an individual’s intention to get vaccinated, including COVID-19 vaccination [11]. Among several useful theories explaining vaccination uptake (e.g., protection motivation theory (PMT [12]), a theory using fear and perceived vulnerability to explain human health behaviors; and health belief models (HBM [13]), a theory using health perceptions to explain human health behaviors), the theory of planned behavior (TPB) [14] serves as the present study’s main framework to investigate an individual’s intention to get COVID-19 vaccinated. The TPB proposes that attitude (i.e., how an individual evaluates a behavior), subjective norm (i.e., how an individual perceived others’ judgement on a behavior), and perceived behavioral control (i.e., how an individual is confident in performing a behavior) are key psychological constructs to explain why an individual performs a specific behavior [14].

More specifically, the efficacy of using TPB on vaccination uptake has been evidenced in research on individuals’ intention to uptake influenza vaccination [15,16], human papillomavirus (HPV) vaccination [17], and more recently COVID-19 vaccination [18]. Moreover, the efficacy of the three main constructs in the TPB (i.e., attitude, subjective norm, and perceived behavioral control) is promising in explaining individuals’ intention to uptake vaccination [19]. One meta-analysis reported the total explained variance of intention to uptake vaccination to be 54% [19]. Although actual behavior (i.e., actual COVID-19 vaccination uptake) is a much more important outcome measure than the intention (i.e., intention to uptake COVID-19 vaccination), the present study focused on intention rather than actual behavior (i.e., COVID-19 vaccination uptake) for the following reason. At the time when data collection took place, the numbers of COVID-19 vaccination doses available were insufficient to cover the entire Iranian population. Therefore, the shortage of COVID-19 vaccination doses may have biased (i.e., underestimated) actual behavior or COVID-19 vaccination uptake. In this regard, using the TPB to explain individuals’ intention to get COVID-19 vaccinated is appropriate.

Taking COVID-19 vaccination uptake as an example in explaining the three constructs, attitude indicates whether an individual judges getting COVID-19 vaccinated as positive or negative. Subjective norm indicates whether an individual receives and perceives others’ (especially those with a close relationship, such as a spouse, or the authorities, such as physicians) opinions on COVID-19 vaccination and therefore changes their belief concerning COVID-19 vaccination. Perceived behavioral control indicates whether an individual perceives whether their control and ability (e.g., time, financial support, and opportunity) assists him or her to get COVID-19 vaccinated. An individual’s intention to get COVID-19 vaccination is subsequently explained by these three aforementioned constructs [18,19]. In summary, the TPB claims that attitude, subjective norm, and perceived behavioral control are important constructs that explain the intention of an individual to perform a human behavior [14]. This can be applied to the behavior of COVID-19 vaccination uptake as well as to the intention to get COVID-19 vaccinated [19,20].

However, some scholars claim that the three main constructs in the TPB are insufficient in explaining complex human behaviors [20]. Therefore, arguments on whether the TPB should be retained in health-related behaviors have been debated [21,22,23,24] and the notion of extending the TPB has been generally acceptable among behavioral researchers. Therefore, the present study extended the TPB with the incorporation of another two important factors (fear and perceived vulnerability) proposed by PMT [25]. The PMT claims that perceived vulnerability (e.g., how an individual perceives the possibility of getting infected) is triggered by fear and then results in an intention for protection (e.g., COVID-19 vaccination uptake) [26,27]. With the consideration of fear and perceived vulnerability (hereafter, the present study uses perceived infectability because only the infectability construct was assessed as part of the vulnerability in the present study), the extended TPB was therefore examined in the present study.

Apart from incorporating fear and perceived infectability into the TPB, to the best of the present authors’ knowledge, there are no large-scale studies discussing the effectiveness of the extended TPB on intention to uptake COVID-19 vaccination. More specifically, the TPB (or part of the TPB) has not been applied to studies with a sample size larger than 10,000 participants. For example, the sample size was 2653 in Zhang et al.’s study [28]; 398 in Shmueli’s study [18]; 374 in Sturman et al.’s study [29]; 2529 in Cordina et al.’s study [30]; and 788 in Guidry et al.’s study [31]. In this regard, the present authors proposed to use a large-scale study incorporating more than 10,000 individuals to fully examine the effectiveness of the extended TPB. Moreover, the large Muslim majority in Iran (99%) could have an effect on their vaccine behavior. More specifically, from religious texts in Islamic books, all of them emphasize observing health tips to prevent disease and prioritize prevention over treatment. Moreover, there have been some concerns about the COVID-19 vaccines in Islamic religion because vaccination is not supposed to be Halal (i.e., lawful in Islamic law). However, religious leaders have had a large influence on Muslims’ willingness to uptake or not uptake vaccination. Therefore, the willingness of vaccination uptake among Iranian Muslims may be different from people with other religious beliefs. As a result, it is important to examine whether the extended TPB can work on such a specific population.

**Figure 1:**
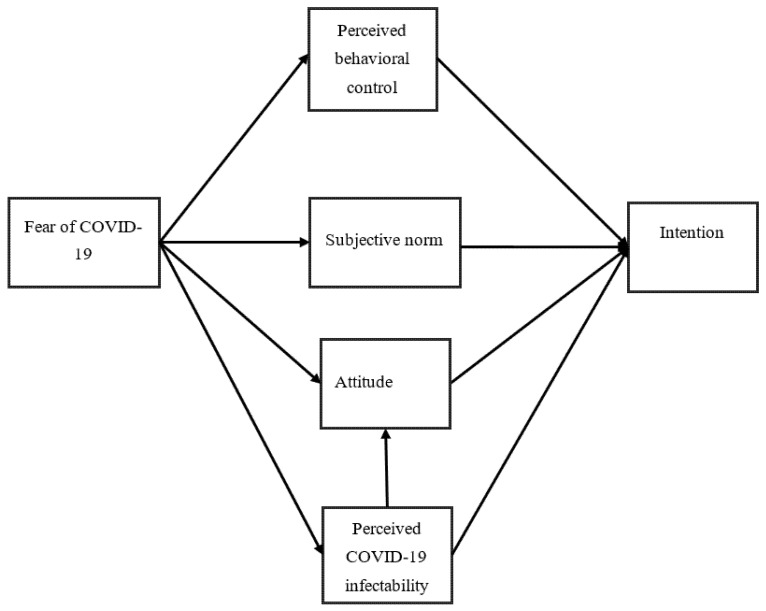
Proposed model to examine intention of COVID-19 vaccination uptake. Perceived behavioral control, subjective norm, attitude, and intention are constructs described in the Theory of Planned Behavior; fear of COVID-19 and perceived COVID-19 infectability are constructs described in Protection Motivation Theory.

The main study aim was to examine the psychological constructs that significantly explained individuals’ intention to get COVID-19 vaccinated with the use of the extended TPB (Figure 1). The specific hypotheses were that (i) fear of COVID-19 would positively explain perceived behavioral control over COVID-19 vaccination uptake, (ii) fear of COVID-19 would positively explain the subjective norm of COVID-19 vaccination uptake, (iii) fear of COVID-19 would positively explain attitude toward COVID-19 vaccination uptake, (iv) fear of COVID-19 would positively explain perceived COVID-19 infectability, (v) perceived COVID-19 infectability would positively explain attitude toward COVID-19 vaccination uptake, (vi) perceived behavioral control over COVID-19 vaccination uptake would positively explain the intention of COVID-19 vaccination uptake, (vii) the subjective norm of COVID-19 vaccination uptake would positively explain the intention of COVID-19 vaccination uptake, (viii) attitude toward COVID-19 vaccination uptake would positively explain the intention of COVID-19 vaccination uptake, and (ix) perceived COVID-19 infectability would positively explain the intention of COVID-19 vaccination uptake.

## 2. Materials and Methods

### 2.1. Participants and Recruitment Procedure

The study population consisted in residents of Qazvin province who lived in cities and villages of the province. Qazvin is one of the cities of Iran, which is located in the central part of this country (50 km northwest of Tehran). According to the latest census conducted in 2018, the population of the province comprised 1,273,761 individuals, with 51% being male. The vaccination policy in Iran comprised the formulation of a priority list for the whole country’s population in 2021. The first priority cohorts included frontline healthcare workers, workers and residents in elderly centers, and veterans. The second priority cohorts were older adults aged over 80 years. At the time of writing, Iran was trying to import vaccines from Russia Sputnik V (Gamaleya Research Institute of Epidemiology and Microbiology, Moscow, Russia), China Sinovac (Sinovac Biotech Ltd., Beijing, China), and South Korea AstraZeneca (SK Bioscience, Gyeonggi-do, Korea), as well as using vaccines under COVID-19 Vaccines Global Access (COVAX).

To collect the data, the study utilized multistage stratified cluster sampling. More specifically, Qazvin Province was stratified into 70 strata. From each stratum, a number of centers were randomly selected from health centers in each stratum based on the size of population. From each health center, families were randomly selected from the list of families in the health centers. Twenty well-trained interviewers then contacted the selected families. The study was then described and their willingness to participate in the study was checked. After participant agreement, the interviewer visited them at home to describe more about the study and to administer the survey. The participation rate was 78% and there was no obvious pattern indicating selection bias across the strata. Therefore, the present study obtained a representative sample of the general adult population in Qazvin. Initially, six cities in Qazvin province (i.e., Qazvin, Takestan, Avaj, Alborz, Buin Zahra, and Abyek), were considered as clusters. Then, the stratification was carried out in each city based on urban and rural areas. Finally, a number of centers were randomly selected from health centers in each urban and rural area and the list of families covered by them were then randomly selected (based on the size of population). Several well-trained interviewers visited the eligible participant’s home to conduct an in-person survey in the period between 19 February and 9 April 2021.

The inclusion criteria for the present study’s participants comprised (i) adult residents (aged 18 years or above) in Qazvin province and (ii) those who were willing to participate in the study. The exclusion criteria were those who were guests and tourists in Iran. All procedures conducted were approved by the Ethics Committee of Qazvin University of Medical Sciences (IR.QUMS.REC.1399.418). Written Informed consent was obtained from all study participants.

### 2.2. Measures

#### 2.2.1. Fear of COVID-19

Fear of COVID-19 was assessed using the Fear of COVID-19 Scale (FCV-19S). The FCV-19S is a well validated instrument that uses seven items to assess an individual’s fear of COVID-19. All the items were assessed using a five-point Likert scale and a higher score indicates a higher level of fear of COVID-19. Prior studies have shown that the FCV-19S has satisfactory psychometric properties in different language versions, including the Persian version [32,33,34]. In the present sample, the internal consistency (α = 0.88), composite reliability (CR = 0.87), and average variance extracted (AVE = 0.48) were acceptable.

#### 2.2.2. Perceived COVID-19 Infectability

Perceived COVID-19 infectability was assessed using five items (sample item: “My immune system protects me from COVID-19 that other people get”). The five items were assessed in the Persian language utilizing a five-point Likert scale. A higher score indicates a higher level of perceived COVID-19 infectability. In the present sample, the internal consistency (α = 0.70), composite reliability (CR = 0.67), and average variance extracted (AVE = 0.30) were acceptable.

#### 2.2.3. Perceived Behavioral Control over COVID-19 Vaccination

Perceived behavioral control over COVID-19 vaccination was assessed using two items (sample item: “I have resources, time and opportunities to get COVID-19 vaccination”). The two items were assessed in the Persian language utilizing a five-point Likert scale. A higher score indicates a higher level of perceived behavioral control over COVID-19 vaccination. In the present sample, the internal consistency (α = 0.75), composite reliability (CR = 0.76), and average variance extracted (AVE = 0.61) were acceptable.

#### 2.2.4. Subjective Norm of COVID-19 Vaccination

Subjective norm of COVID-19 vaccination was assessed using two items (sample item: “Most people who are important to me would want me to get COVID-19 vaccination”). The two items were assessed in the Persian language utilizing a five-point Likert scale. A higher score indicates a higher level of subjective norm of COVID-19 vaccination. In the present sample, the internal consistency (α = 0.89), composite reliability (CR = 0.88), and average variance extracted (AVE = 0.79) were acceptable.

#### 2.2.5. Attitude toward COVID-19 Vaccination

Attitude toward COVID-19 vaccination was assessed using six items (sample item: “For me, getting the COVID-19 vaccination would be extremely bad (scoring 1)/extremely good (scoring 5)). The six items were assessed in the Persian language utilizing a five-point Likert scale. A higher score indicates a higher level of subjective norm of COVID-19 vaccination. In the present sample, the internal consistency (α = 0.94), composite reliability (CR = 0.94), and average variance extracted (AVE = 0.72) were acceptable.

#### 2.2.6. Intention to Get COVID-19 Vaccinated

Intention to get COVID-19 vaccinated was assessed using two items (sample item: “I am willing to get COVID-19 vaccination”). The two items were assessed in the Persian language utilizing a five-point Likert scale. A higher score indicates a higher level of intention to get COVID-19 vaccinated. In the present sample, the internal consistency (α = 0.92), composite reliability (CR = 0.91), and average variance extracted (AVE = 0.84) were acceptable.

### 2.3. Data Analysis

Descriptive statistics were firstly carried out to understand the present sample’s demographic characteristics and the scores of the tested constructs (including fear of COVID-19, perceived infectability, and the TPB constructs of attitude, subjective norm, and perceived behavioral control). Pearson correlations were then used to understand the bivariate correlations between the tested constructs. Structural equation modeling (SEM) with the maximum likelihood estimator was performed to examine the model that incorporated fear of COVID-19 and perceived infectability into the TPB. More specifically, perceived infectability, perceived behavioral control, subjective norms, and attitude were hypothesized as the mediators in the relationship between fear of COVID-19 and intention to get COVID-19 vaccinated (see Figure 1 for the illustrated proposed model). In order to have a better understanding regarding the additional explained variance in perceived COVID-19 infectability and fear of COVID-19, nested models were evaluated. Following this, invariance using multigroup SEM was tested for the final supported model across gender, living area, and age. Bias-corrected bootstrap test with 10,000 bootstrapping samples was used to examine whether the mediated effects exist. Furthermore, the following fit indices were used to examine whether the collected data fitted well with the proposed model: comparative fit index (CFI) > 0.95, Tucker-Lewis index (TLI) > 0.95, root mean square error of approximation (RMSEA) < 0.06, and standardized root mean square residual (SRMR) < 0.08 [35]. The SEM was done using the IBM AMOS 24.0 (IBM SPSS, Chicago, IL).

## 3. Results

The characteristics of the large sample (*n* = 10,843) are reported in Table 1. In brief, the mean age of the participants was 35.54 years (SD = 12.00); slightly over one-third of the participants were males (*n* = 4092; 37.7%); nearly three-quarters of the participants were currently married (*n* = 8092; 74.6%); slightly over three-quarters of the participants resided in a city area (*n* = 8186; 75.5%); and the top three educational levels were having a university degree (*n* = 4230; 39.0%), having a diploma degree (*n* = 2761; 25.5%), and completing secondary high school education (*n* = 1540; 14.2%). Moreover, all the participants in the present study were Muslim.

Table 2 reports the bivariate correlations between the fear of COVID-19, perceived infectability, and TPB constructs. All the bivariate correlations were significant (*p* < 0.01) with the magnitudes ranging between 0.172 and 0.765. Moreover, Table 3 reports the mean (SD) scores of the fear of COVID-19, perceived infectability, and TPB constructs (i.e., attitude, subjective norm, perceived behavioral control, and intention).

Table 3 further reports the item properties for all the tested constructs (i.e., fear of COVID-19, perceived infectability, and TPB constructs). All the items had moderate to strong factor loadings. More specifically, factor loadings (i.e., how an item was associated with a latent construct) for attitude construct were between 0.794 and 0.909; for subjective norm were 0.881 and 0.898; for perceived behavioral control were 0.720 and 0.837; for intention were 0.907 and 0.927; for perceived COVID-19 infectability were between 0.384 and 0.703; for fear of COVID-19 were between 0.631 and 0.765.

**Figure 2:**
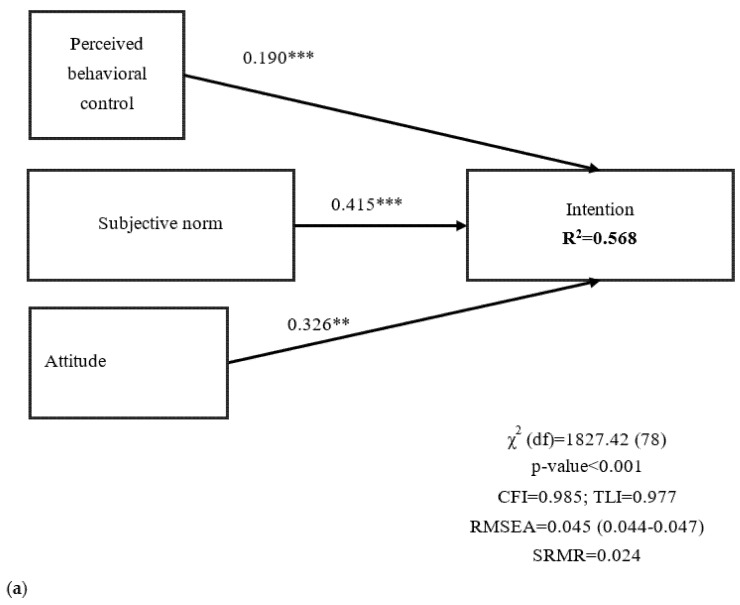

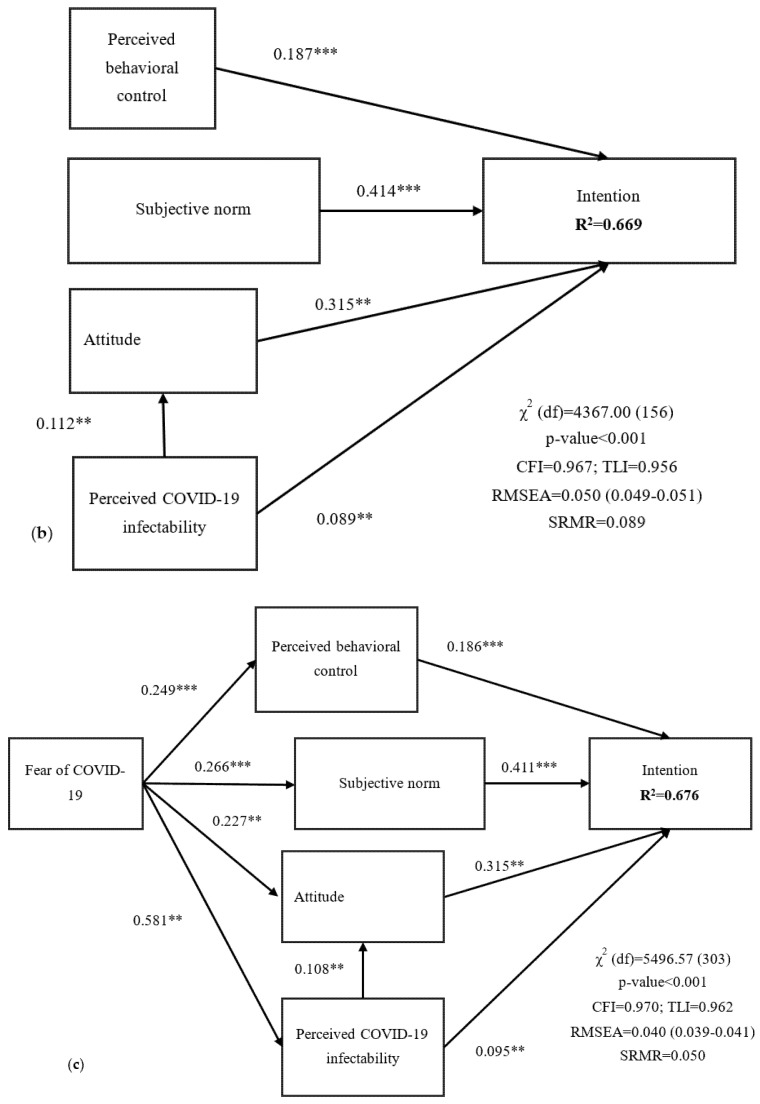
(**a**) Findings in the proposed model without perceived COVID-19 infectability and fear of COVID-19; (**b**) Findings in the proposed model without fear of COVID-19; (**c**) Findings in the proposed model; all the constructs together explained 67.6% of the intention to get COVID-19 vaccinated. Perceived behavioral control, subjective norm, attitude, and intention are constructs described in Theory of Planned Behavior; fear of COVID-19 and perceived COVID-19 infectability are constructs described in Protection Motivation Theory. ** *p* < 0.01; *** *p* < 0.001; CFI = comparative fit index; TLI = Tucker-Lewis index; RMSEA = root mean square error of approximation; SRMR = standardized root-mean-square residual.

All the nested models had satisfactory fit statistics, except for the significant χ^2^ test, indicating that the TPB and extended TPB were both supported. Moreover, nested models showed that perceived infectability added 10.1% of explained variance on intention to uptake COVID-19 vaccination. Fear of COVID-19 further added 0.7% of explained variance (Figure 2). When scrutinizing the proposed final model that incorporates fear of COVID-19 and perceived COVID-19 infectability with the TPB, it had a good fit for all the fit indices but a significant χ^2^ test (χ^2^ = 5496.57; *p* < 0.001). The satisfactory fit indices of the final model were CFI = 0.970, TLI = 0.962, RMSEA = 0.040 (90% CI 0.039, 0.041), and SRMR = 0.050. Moreover, the proposed final model’s path coefficients were all significant (Figure 2c), indicating that perceived behavioral control, subjective norm, attitude, and perceived COVID-19 infectability significantly explained an individual’s intention to get COVID-19 vaccinated (Figure 2). The final model showed that the path coefficients were invariant across age, gender, and living place (Table 4). Mediation analyses further indicated that perceived COVID-19 infectability and TPB constructs were all significant mediators in the relationship between fear of COVID-19 and intention to get COVID-19 vaccinated (standardized coefficient = 0.302; coefficient = 0.369; SE = 0.007; 95% CI from the bias-corrected bootstrap test = 0.278, 0.327; *p* < 0.001).

## 4. Discussion

To the best of the present authors’ knowledge, the present research is the first large-scale study presenting empirical evidence of using extended TPB on individuals’ intention to get COVID-19 vaccinated. The findings indicated that incorporating fear of COVID-19 and perceived COVID-19 infectability into the TPB was effective in explaining Iranian people’s intention to get COVID-19 vaccinated. The proposed model was supported by the data, and the nine hypotheses in the present study were all supported. More specifically, (i) fear of COVID-19 positively explained perceived behavioral control over COVID-19 vaccination uptake, (ii) fear of COVID-19 positively explained subjective norm of COVID-19 vaccination uptake, (iii) fear of COVID-19 positively explained attitude toward COVID-19 vaccination uptake, (iv) fear of COVID-19 positively explained perceived COVID-19 infectability, (v) perceived COVID-19 infectability positively explained attitude toward COVID-19 vaccination uptake, (vi) perceived behavioral control over COVID-19 vaccination uptake positively explained the intention of COVID-19 vaccination uptake, (vii) subjective norm of COVID-19 vaccination uptake positively explained the intention of COVID-19 vaccination uptake, (viii) attitude toward COVID-19 vaccination uptake positively explained the intention of COVID-19 vaccination uptake, and (ix) perceived COVID-19 infectability positively explained the intention of COVID-19 vaccination uptake. By 27 April 2021, the uptake rate of COVID-19 vaccination in Iran was 0.2% [36] although one Iranian study reported that 65.7% Iranians from 370 online respondents were willing to pay for COVID-19 vaccination in November 2020 [37]. In this regard, understanding the psychological factors that explain the intention to get COVID-19 vaccinated among Iranians is important for authorities and healthcare providers to boost the uptake rate. Consequently, the infection rate of COVID-19 can be effectively controlled.

The effectiveness of TPB in explaining human behaviors has been widely studied [38,39,40] and the present study’s findings extend the effectiveness of TPB to the intention of COVID-19 vaccination uptake. That is, the present study found that attitude, subjective norms, and perceived behavioral control in the TPB were all significant factors explaining intention of COVID-19 vaccination uptake. This finding corresponds well with what prior TPB studies on vaccination uptake have found [18,28,29,30,31]. For example, Guidry et al. found that American adults who were more positive toward COVID-19 vaccination and had higher levels of subjective norms held greater intention to get COVID-19 vaccinated [31]. Zhang et al. reported that mainland Chinese factor workers who had greater perceived behavioral control possessed a higher level of intention to get COVID-19 vaccinated [28]. Shmueli recently incorporated the TPB with the HBM to explain the intention to get COVID-19 vaccinated among Israeli adults and found the incorporation of TPB and HBM was effective [18].

Moreover, the incorporation of fear and perceived COVID-19 infectability from the PMT [25,26] supplements the usefulness of the TPB. Therefore, healthcare providers may use fear and perceived COVID-19 infectability (e.g., distributing the true information that COVID-19 infection has 2% of mortality rate and the prevalence of COVID-19 confirmed cases is high) to improve an individual’s intention to uptake COVID-19 vaccination. The present study found that perceived COVID-19 infectability was a significant factor in explaining individuals’ intention to get COVID-19 vaccinated. However, the findings contradict those of Wang et al. [27]. More specifically, they found that perceived vulnerability (i.e., a construct similar to the present study’s perceived infectability) was not associated with individuals’ intention to get COVID-19 vaccinated. A potential explanation is the difference in studied populations between the two studies. Wang et al. tested the association between perceived COVID-19 vulnerability and intention to get COVID-19 vaccinated among mainland Chinese university students and the present study tested this association among the Iranian general population [27]. Because the infection severities of COVID-19 are different in the two countries (i.e., Iran has a more serious COVID-19 situation than mainland China), it is possible that the participants in Wang et al.’s study had low levels of perceived vulnerability which subsequently contributed to the nonsignificant association with intention to get vaccinated [27].

However, the present study’s findings echo those from many studies that fear of COVID-19 is associated with intention to get COVID-19 vaccinated [41,42,43]. Moreover, the present findings provide evidence showing a potential mechanism as to why the fear of COVID-19 is associated with intention to get COVID-19 vaccinated. More specifically, fear of COVID-19 may trigger individual’s psychological factors of perceived behavioral control over COVID-19 vaccination uptake, subjective norm of COVID-19 vaccination uptake, attitude to COVID-19 vaccination uptake, and perceived COVID-19 infectability. These psychological factors in turn are likely to contribute to individuals’ intentions to get COVID-19 vaccinated. This psychological mechanism also works in the Iranian population who have a strong belief in Muslim religion.

There are some limitations in the present study. First, the present study used self-reports as the primary data collection method. Therefore, the present study has method biases related to this type of data collection, such as single-rater bias, recall bias, and social desirability bias. It should also be noted that the item “I have resources, time and opportunities to get COVID-19 vaccination” used in assessing perceived behavioral control construct is a triple-barreled question, which may not accurately assess the situation of an individual (e.g., someone has adequate time but no resources may have difficulties in answering this item). Therefore, perceived behavioral control assessed in the present study may be somewhat biased. Second, the study design was cross-sectional and the causal relationships among the studied constructs (i.e., fear of COVID-19, perceived COVID-19 infectability, and TPB constructs) cannot be determined. Although the model proposed in the present study was guided by the TPB and PMT, the present findings only weakly support COVID-19 vaccination uptake intention. Future studies are therefore needed to investigate whether the changes in the studied constructs are associated. Third, the present study did not assess barriers relating to unwillingness to get COVID-19 vaccinated. Therefore, it is unclear if there are some important factors that may prevent individuals from getting COVID-19 vaccinated. Future studies are therefore needed to explore barriers to COVID-19 vaccination. Fourth, the present study did not assess the full concept of the perceived vulnerability in PMT because only the infectability construct was assessed as part of the vulnerability in the present study. This may thus lower the efficacy of the proposed model in the present study. Fifth, the present study findings may not be generalizable to other countries given that the severities of COVID-19 outbreak are different across other countries, cultures, and regions. With the different situations of COVID-19 outbreak, the intention to get COVID-19 vaccinated may not be the same across geographical regions. For example, Iranians were found to have a higher level of fear toward COVID-19 when compared with those residing in other countries [34]. Therefore, the high level of fear may strengthen the associations between fear of COVID-19 and TPB constructs. However, in order to effectively control COVID-19 infection, efforts from all countries worldwide should be worked out in a coordinated framework. Therefore, the specific findings from the Iranian population are arguably important in controlling the spread of worldwide COVID-19 infection. Following this limitation, given that the present study recruited mostly Muslim individuals, the findings cannot necessarily be generalized to other religious populations (e.g., Christianity and Buddhism). It is possible that religiosity or cultural background may impact on COVID-19 vaccination uptake intention or behavior. Therefore, future studies are encouraged to examine this issue further. Finally, the present study did not assess the actual behavior of COVID-19 vaccination uptake because this information might have been biased due to the shortage of vaccination doses in Iran. Individuals’ intention does not indicate their actual behavior in getting COVID-19 vaccinated. Therefore, future studies are encouraged to collect behavior information when the Iranian government has sufficient COVID-19 vaccination doses.

## 5. Conclusions

The present study demonstrated that the TPB incorporated with the fear of COVID-19 and perceived COVID-19 infectability can be used to design effective programs that will improve COVID-19 vaccination uptake rate among the Iranian population. For example, Iranian authorities and healthcare providers may advocate the beneficial effects of COVID-19 vaccination (i.e., improving the attitude construct in TPB because attitude was found to be significantly associated with intention to get COVID-19 vaccinated) and provide accurate facts regarding the adverse effects of COVID-19 vaccination uptake (i.e., improving the subjective norm construct in TPB because the subjective norm was found to be significantly associated with intention to get COVID-19 vaccinated). More specifically, positive advertisements for COVID-19 vaccines, showing celebrities and important authorities getting vaccinated, may increase subjective norms and subsequently improve the willingness of COVID-19 vaccination uptake among Iranian general population. With such promotion, individuals’ subjective norm and attitude towards COVID-19 vaccination uptake may be improved. Moreover, governments may consider several methods to deliver COVID-19 vaccination among individuals residing in distant areas to improve the perceived behavioral control of such individuals. Because the present findings indicated that perceived behavioral control was associated with intention to get COVID-19 vaccinated, improved perceived behavioral control may further increase the intention to get COVID-19 vaccinated for these individuals. As a result, the intention to get COVID-19 vaccinated may be elevated with the aforementioned efforts.

## Figures and Tables

**Table 1:** The demographic characteristics of the participants included in this study.

Demographic Variable	Mean ± SD or *n* (%)
**Age**	35.54 ± 12.00
**Gender**	
Male	4092 (37.7%)
**Educational levels**	
University	4230 (39.0%)
Diploma	2761 (25.5%)
High school	974 (9.0%)
Secondary school	1540 (14.2%)
Primary school	986 (9.1%)
Illiterate	352 (3.2%)
**Marital status**	
Married	8092 (74.6%)
Single	2751 (25.4%)
**Accommodation**	
City	8186 (75.5%)
Rural	2656 (24.5%)

SD = Standard deviation.

**Table 2:** The correlations of theory of planned behavior variables.

Variable	1	2	3	4	5	6
**1. Fear of COVID-19**	1					
**2. Perceived infectability**	0.419 **	1				
**3. Perceived behavioral control**	0.172 **	0.241 **	1			
**4. Subjective norms**	0.213 **	0.318 **	0.487 **	1		
**5. Attitude**	0.232 **	0.345 **	0.492 **	0.765 **	1	
**6. Intention**	0.219 **	0.325 **	0.523 **	0.686 **	0.740 **	1

** = *p* < 0.01.

**Table 3:** Items for Study Measures with Descriptive Statistics, Reliability Coefficients, Factor Loadings, and Average Variance Extracted Statistics.

Construct; Mean (SD)	Measurement Item	λ	α	CR	AVE
**Fear of COVID-19;** **21.12 (6.94)**			0.88	0.87	0.48
	I am most afraid of coronavirus-19	0.648			
	It makes me uncomfortable to think about coronavirus-19	0.742			
	My hands become clammy when I think about coronavirus-19	0.631			
	I am afraid of losing my life because of coronavirus-19	0.735			
	When watching news and stories about coronavirus-19 on social media, I become nervous or anxious	0.765			
	I cannot sleep because I’m worrying about getting coronavirus-19	0.650			
	My heart races or palpitates when I think about getting coronavirus-19	0.670			
**Perceived COVID-19 infectability;** **3.12 (1.10)**			0.70	0.67	0.30
	If a COVID-19 patient is “going around in his/her immediate locality”, I will get it	0.703			
	My past experiences make me believe I am not likely to get COVID-19 even when my friends are sick	0.384			
	In general, I am very susceptible to colds, flu, COVID-19 and other infectious diseases	0.529			
	I am unlikely to catch a cold, flu, COVID-19 or other illness, even if it is “going around in the immediate locality”	0.457			
	My immune system protects me from COVID-19 that other people get	0.615			
**Perceived Behavioral Control;** **3.84 (1.05)**			0.75	0.76	0.61
	Whether or not I get COVID-19 vaccination is completely up to me.	0.720			
	I have resources, time and opportunities to get COVID-19 vaccination	0.837			
**Subjective norms;** **3.83 (1.10)**	*Most people who are important to me would…*		0.89	0.88	0.79
	want me to get COVID-19 vaccination	0.881			
	think I should get COVID-19 vaccination	0.898			
**Attitude;** **3.86 (0.96)**	*For me, getting the COVID-19 vaccination would be …*		0.94	0.94	0.72
	extremely bad (1)/extremely good (5)	0.816			
	extremely undesirable (1)/extremely desirable (5)	0.879			
	extremely unimportant (1)/extremely important (5)	0.909			
	extremely useless (1)/extremely useful (5)	0.808			
	extremely unfavorable (1)/extremely favorable (5)	0.809			
	extremely harmful (1)/extremely beneficial (5)	0.794			
**Intentions;** **3.84 (1.10)**			0.92	0.91	0.84
	I am willing to get COVID-19 vaccination	0.927			
	I want to get COVID-19 vaccination	0.907			

Note. λ = Standardized factor loading from structural equation model; α = Cronbach’s alpha reliability coefficient; CR = Composite reliability; AVE = Average variance extracted from structural equation model; SD = Standard deviation.

**Table 4:** Invariance evaluation across age, gender, and living place through multigroup structural equation modeling.

Model and Comparisons	Fit Statistics						
	χ^2^ (df)	∆χ^2^ (∆df)	CFI	∆CFI	TLI	∆TLI	RMSEA
**Gender (male vs. female)**							
M1: Unconstrained	5257.24 (460) *	-	0.971	-	0.965	-	0.031
M2: Measurement weights	5305.79 (478) *	45.552(18) *	0.971	0	0.967	0.002	0.031
M3: Measurement intercepts	5889.68 (502) *	632.44(42) *	0.968	−0.002	0.964	−0.001	0.031
M4: Structural weights	5909.02 (512) *	651.78(52) *	0.968	−0.002	0.965	0.00	0.031
M5: Structural Covariances	5914.90 (513) *	657.66(53) *	0.968	−0.002	0.965	0.00	0.031
M6: Structural Residuals	6009.70 (521) *	752.46(61) *	0.967	−0.003	0.965	0.00	0.031
M7: Measurement Residuals	6726.63 (554) *	1469.39(94) *	0.963	−0.008	0.963	0.002	0.032
**Age (above 35 years vs. below 35 years)**							
M1: Unconstrained	5307.66 (460) *	-	0.971	-	0.965	-	0.031
M2: Measurement weights	5365.10 (478) *	57.44(18) *	0.971	0	0.966	0.001	0.031
M3: Measurement intercepts	5606.77 (502) *	299.11(42) *	0.969	−0.002	0.966	0.001	0.031
M4: Structural weight	5610.44 (512) *	302.78(52)	0.970	−0.001	0.967	0.002	0.030
M5: Structural Covariances	5610.57 (513) *	302.91(53) *	0.970	−0.001	0.967	0.002	0.030
M6: Structural Residuals	5706.17 (521) *	398.51(61) *	0.969	−0.001	0.967	0.002	0.030
M7: Measurement Residuals	6263.78 (554) *	956.12(94) *	0.966	−0.005	0.966	0.001	0.031
**Living (city vs. rural)**							
M1: Unconstrained	5214.69 (460) *	-	0.972	-	0.966	-	0.031
M2: Measurement weights	5259.74 (478) *	45.05 (18) *	0.971	−0.001	0.967	0.001	0.030
M3: Measurement intercepts	5419.85 (502) *	205.16(42) *	0.971	−0.001	0.968	0.002	0.030
M4: Structural weights	5450.73 (512) *	236.03(52) *	0.970	−0.002	0.968	0.002	0.030
M5: Structural Covariances	5450.74 (513) *	236.05(53) *	0.970	−0.002	0.968	0.002	0.030
M6: Structural Residuals	5508.89 (521) *	294.20(61) *	0.970	−0.002	0.968	0.002	0.030
M7: Measurement Residuals	5692.11 (554) *	477.42(94) *	0.969	−0.003	0.969	0.003	0.029

* *p* < 0.05. CFI = comparative fit index; TLI = Tucker-Lewis index; RMSEA = root mean square error of approximation.

## Data Availability

Data availability is in a publicly accessible repository. The data presented in this study are openly available in Harvard Dataverse at https://doi.org/10.7910/DVN/IETC88, reference number IETC88.

## References

[1] Crayne M.P. (2020). The traumatic impact of job loss and job search in the aftermath of COVID-19. Psychol. Trauma.

[2] McIntyre R.S., Lee Y. (2020). Preventing suicide in the context of the COVID-19 pandemic. World J. Psychiatry.

[3] Oksanen A., Kaakinen M., Latikka R., Savolainen I., Savela N., Koivula A. (2020). Regulation and trust: 3-month follow-up study on COVID-19 mortality in 25 European countries. JMIR Public Health Surveill..

[4] Torales J., O’Higgins M., Castaldelli-Maia J.M., Ventriglio A. (2020). The outbreak of COVID-19 coronavirus and its impact on global mental health. Int. J. Soc. Psychiatry.

[5] WHO Director-General’s Opening Remarks at the Media Briefing on COVID-19—11 March 2020. https://www.who.int/director-general/speeches/detail/who-director-general-s-opening-remarks-at-the-media-briefing-on-covid-19---11-march-2020.

[6] Rundle A.G., Park Y., Herbstman J.B., Kinsey E.W., Wang Y.C. (2020). COVID-19 related school closings and risk of weight gain among children. Obesity.

[7] (2021). DRAFT Landscape of COVID-19 Candidate Vaccines. https://www.who.int/publications/m/item/draft-landscape-of-covid-19-candidate-vaccines.

[8] Hotez P. (2019). America and Europe’s new normal: The return of vaccine-preventable diseases. Pediatr. Res..

[9] Dubé E., Vivion M., MacDonald N.E. (2015). Vaccine hesitancy, vaccine refusal and the anti-vaccine movement: Influence, impact and implications. Expert. Rev. Vaccines.

[10] Trogen B., Oshinsky D., Caplan A. (2020). Adverse consequences of rushing a SARS-CoV-2 vaccine: Implications for public trust. JAMA.

[11] Prestwich A., Webb T.L., Conner M. (2015). Using theory to develop and test interventions to promote changes in health behaviour: Evidence, issues, and recommendations. Curr. Opin. Psychol..

[12] Rogers R.W., Cacioppo J., Petty R. (1983). Cognitive and physiological processes in fear appeals and attitude change: A revised theory of protection motivation. Social Psychophysiology.

[13] Rosenstock I.M. (1966). Why people use health services. Milbank Mem. Fund Q..

[14] Ajzen I. (1991). The theory of planned behavior. Organ Behav. Hum. Decis. Process.

[15] Schmid P., Rauber D., Betsch C., Lidolt G., Denker M.L. (2017). Barriers of influenza vaccination intention and behavior—A systematic review of influenza vaccine hesitancy, 2005–2016. PLoS ONE.

[16] Wu A.M.S., Lau J.T.F., Ma Y.L., Cheng K.M., Lau M.M.C. (2020). A longitudinal study using parental cognitions based on the theory of planned behavior to predict childhood influenza vaccination. J. Infect. Public Health.

[17] Catalano H.P., Knowlden A.P., Birch D.A., Leeper J.D., Paschal A.M., Usdan S.L. (2017). Using the theory of planned behavior to predict HPV vaccination intentions of college men. J. Am. Coll. Health.

[18] Shmueli L. (2021). Predicting intention to receive COVID-19 vaccine among the general population using the health belief model and the theory of planned behavior model. BMC Public Health.

[19] Xiao X., Wong R.M. (2020). Vaccine hesitancy and perceived behavioral control: A meta-analysis. Vaccine.

[20] Sniehotta F.F., Presseau J., Araújo-Soares V. (2014). Time to retire the theory of planned behaviour. Health Psychol. Rev..

[21] Ajzen I. (2015). The theory of planned behaviour is alive and well, and not ready to retire: A commentary on Sniehotta, Presseau, and Araújo-Soares. Health Psychol. Rev..

[22] Armitage C.J. (2015). Time to retire the theory of planned behaviour? A commentary on Sniehotta, Presseau and Araújo-Soares. Health Psychol. Rev..

[23] Conner M. (2015). Extending not retiring the theory of planned behaviour: A commentary on Sniehotta, Presseau and Araújo-Soares. Health Psychol. Rev..

[24] Ogden J. (2015). Time to retire the theory of planned behaviour? One of us will have to go! A commentary on Sniehotta, Presseau and Araújo-Soares. Health Psychol. Rev..

[25] Roger R.W. (1975). A protection motivation theory of fear appeals and attitude change. J. Psychol..

[26] Ling M., Kothe E.J., Mullan B.A. (2019). Predicting intention to receive a seasonal influenza vaccination using Protection Motivation Theory. Soc. Sci. Med..

[27] Wang P.-W., Ahorsu D.K., Lin C.-Y., Chen I.-H., Yen C.-F., Kuo Y.-J., Griffiths M.D., Pakpour A.H. (2021). Motivation to have COVID-19 vaccination explained Using an extended protection motivation theory among university students in China: The role of information sources. Vaccines.

[28] Zhang K.C., Fang Y., Cao H., Chen H., Hu T., Chen Y., Zhou X., Wang Z. (2021). Behavioral intention to receive a COVID-19 vaccination among Chinese factory workers: Cross-sectional online survey. J. Med. Internet Res..

[29] Sturman D., Auton J.C., Thacker J. (2021). Knowledge of social distancing measures and adherence to restrictions during the COVID-19 pandemic. Health Promot. J. Aust..

[30] Cordina M., Lauri M.A., Lauri J. (2021). Attitudes towards COVID-19 vaccination, vaccine hesitancy and intention to take the vaccine. Pharm. Pract..

[31] Guidry J.P.D., Laestadius L.I., Vraga E.K., Miller C.A., Perrin P.B., Burton C.W., Ryan M., Fuemmeler B.F., Carlyle K.E. (2021). Willingness to get the COVID-19 vaccine with and without emergency use authorization. Am. J. Infect. Control.

[32] Ahorsu D.K., Lin C.-Y., Imani V., Saffari M., Griffiths M.D., Pakpour A.H. (2020). Fear of COVID-19 Scale: Development and initial validation. Int. J. Ment. Health Addict..

[33] Chang K.-C., Hou W.-L., Pakpour A.H., Lin C.-Y., Griffiths M.D. (2020). Psychometric testing of three COVID-19-related scales among people with mental illness. Int. J. Ment. Health Addict..

[34] Lin C.-Y., Hou W.-L., Mamun M.A., da Silva J.A., Broche-Pérez Y., Ullah I., Masuyama A., Wakashima K., Mailliez M., Carre A. (2021). Fear of COVID-19 Scale (FCV-19S) across countries: Measurement invariance issues. Nurs. Open.

[35] Hu L.-T., Bentler P.M. (1999). Cutoff criteria for fit indexes in covariance structure analysis: Conventional criteria versus new alternatives. Struct. Equ. Modeling.

[36] COVID-19 Data. https://github.com/owid/covid-19-data/blob/master/public/data/vaccinations/country_data/Iran.csv.

[37] Adeli O.A., Kah Kashi S.R. (2021). Estimating willingness to pay for the Covid-19 vaccine using the conditional valuation method. J. Iran Inst. Health Sci. Res..

[38] Armitage C.J., Conner M. (2001). Efficacy of the Theory of Planned Behaviour: A meta-analytic review. Br. J. Soc. Psychol..

[39] Hagger M.S., Hamilton K. (2021). Effects of socio-structural variables in the theory of planned behavior: A mediation model in multiple samples and behaviors. Psychol. Health.

[40] Rich A., Brandes K., Mullan B., Hagger M.S. (2015). Theory of planned behavior and adherence in chronic illness: A meta-analysis. J. Behav. Med..

[41] Detoc M., Bruel S., Frappe P., Tardy B., Botelho-Nevers E., Gagneux-Brunon A. (2020). Intention to participate in a COVID-19 vaccine clinical trial and to get vaccinated against COVID-19 in France during the pandemic. Vaccine.

[42] Gagneux-Brunon A., Detoc M., Bruel S., Tardy B., Rozaire O., Frappe P., Botelho-Nevers E. (2021). Intention to get vaccinations against COVID-19 in French healthcare workers during the first pandemic wave: A cross-sectional survey. J. Hosp. Infect..

[43] Qiao S., Tam C.C., Li X. (2020). Risk exposures, risk perceptions, negative attitudes toward general vaccination, and COVID-19 vaccine acceptance among college students in South Carolina. medRxiv.

